# Supplementary figures and images for: Optimal immune specificity at the intersection of host life history and parasite epidemiology
Source: PLoS Comput Biol. 2021 Dec 21;17(12):e1009714. doi: 10.1371/journal.pcbi.1009714 (PMC8730424; doi:10.1371/journal.pcbi.1009714)

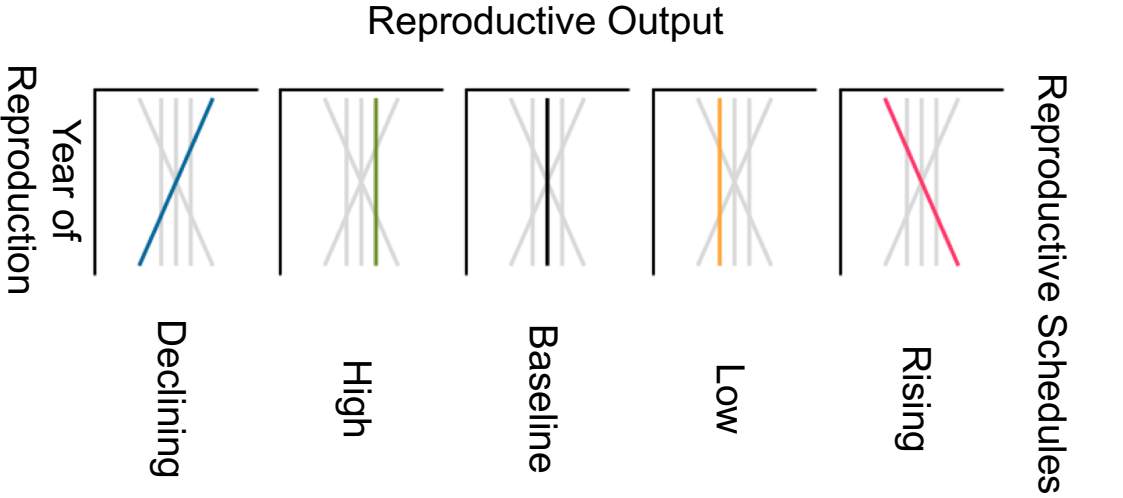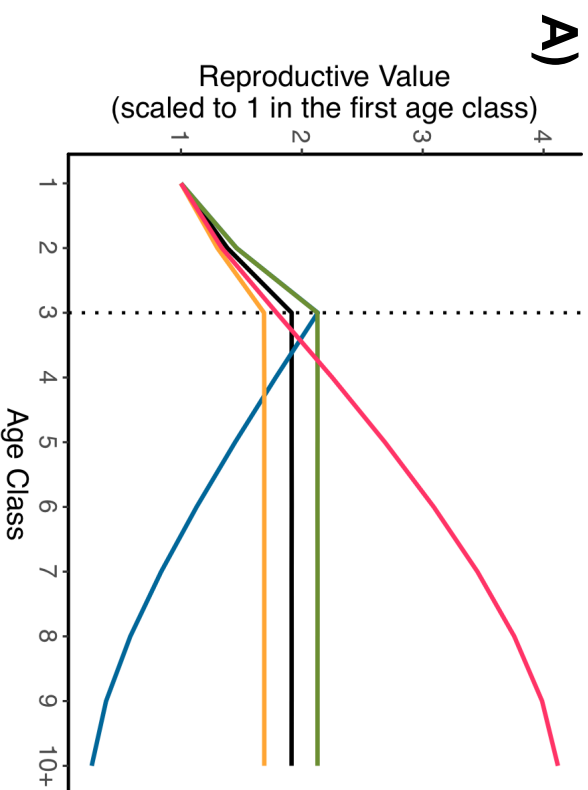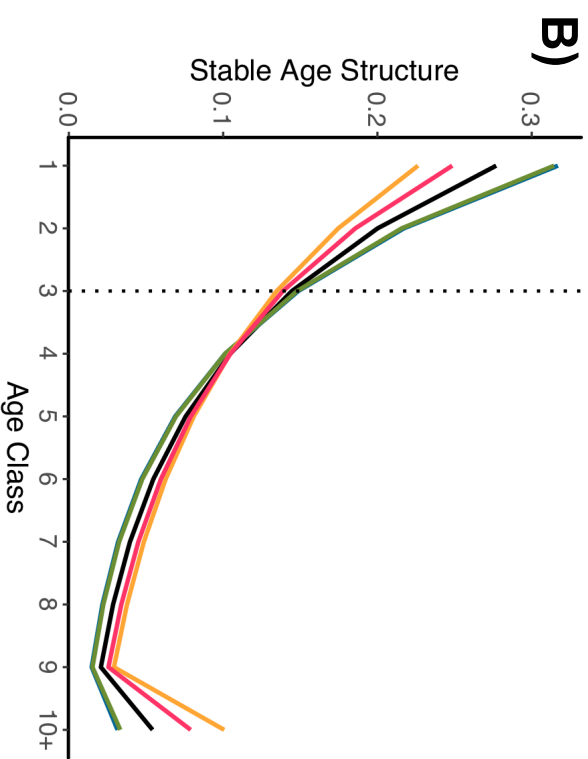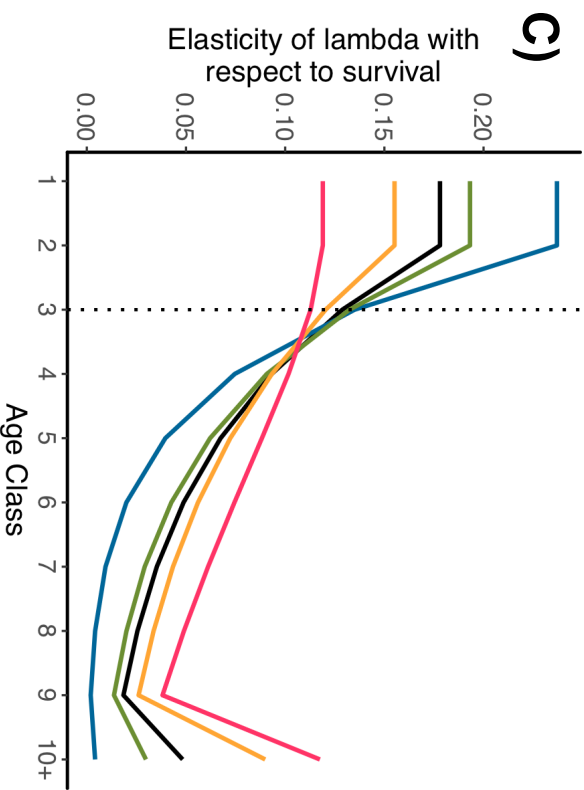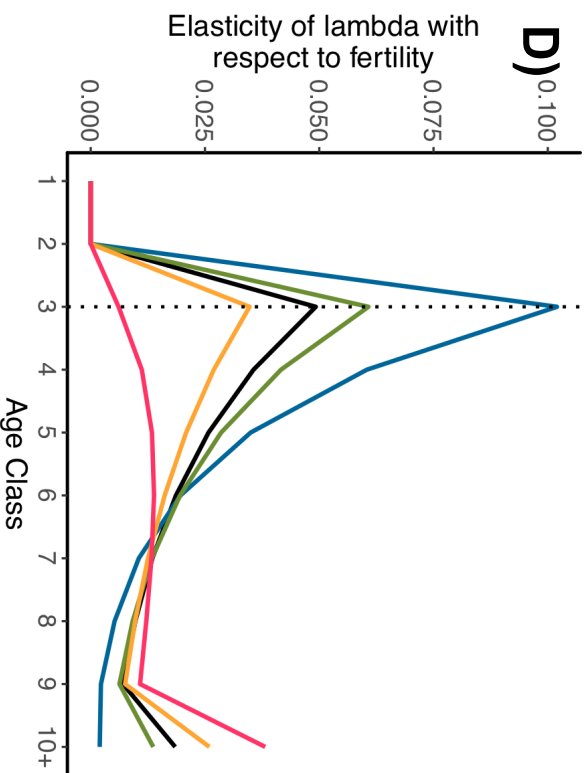

Supplement: S1 Fig — Optimal immune strategies identified in Fig 2 and S1 Table. Dashed line shows age class of reproductive maturity, the third age class. A) Reproductive value distributions, with reproductive value in the first age class defined as 1; B) Stable age structures; C) Elasticities of λ with respect to survival; D) Elasticities of λ with respect to fertility. Infection risk ir drops from 0.6 in pre-reproductive age classes to 0.2 in reproductive age classes. Other parameter values are μb = 0.15, μi = 0.1, μd = 0.3, μdi = 0.01, and γ = 4. (PDF) [file pcbi.1009714.s001.pdf]

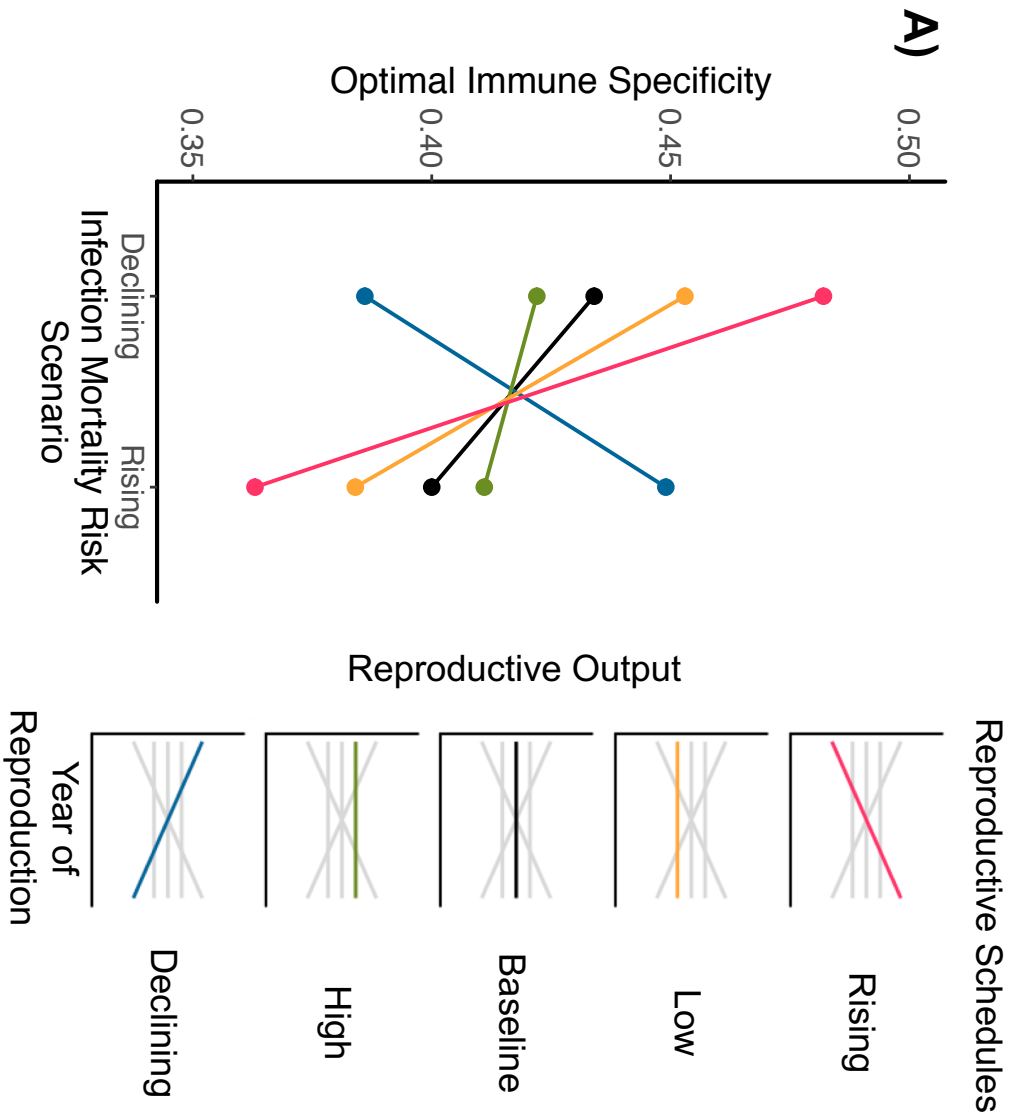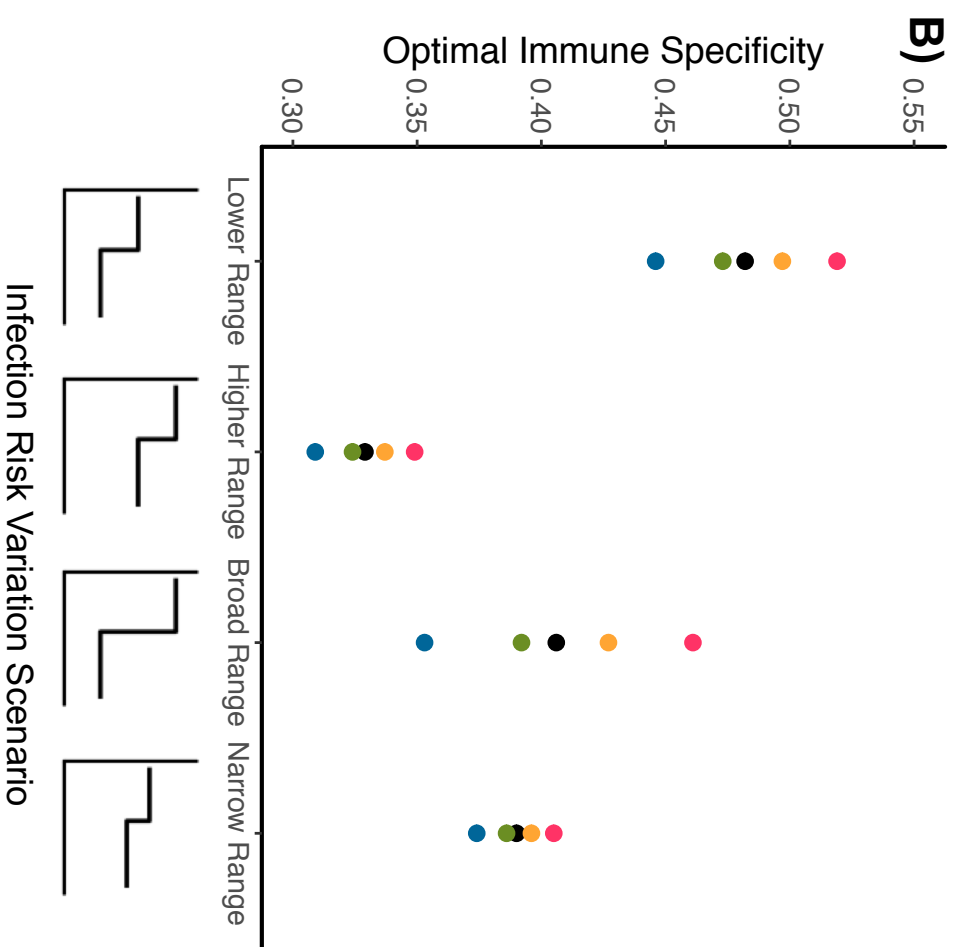

Supplement: S2 Fig — Reproduction begins in the third age class for all schedules. In each scenario, μd starts at one value and drops to a lower value at the third age class. A) The change in optimal immune specificity associated with differences in epidemiological context (i.e. changes in μd, on the x-axis) and reproduction (different points and lines, color-coded at center). Parameter values are μb = 0.15, μi = 0.1, μid = 0.01, ir = 0.4, and γ = 4. In the declining scenario, μd drops with age from 0.6 to 0.2; in the rising scenario, μd increases from 0.2 to 0.6. B) The change in range of optimal specificities associated with different reproductive demographies associated with different magnitudes of variation in decline of infection mortality risk μd with age. Parameter values are μb = 0.15, μi = 0.1, μid = 0.01, ir = 0.4, and γ = 4. In the lower range scenario, μd declines from 0.45 to 0.2; in the higher range, from 0.7 to 0.45; in the broad range, from 0.7 to 0.2; in the narrow range, from 0.525 to 0.375. (PDF) [file pcbi.1009714.s002.pdf]

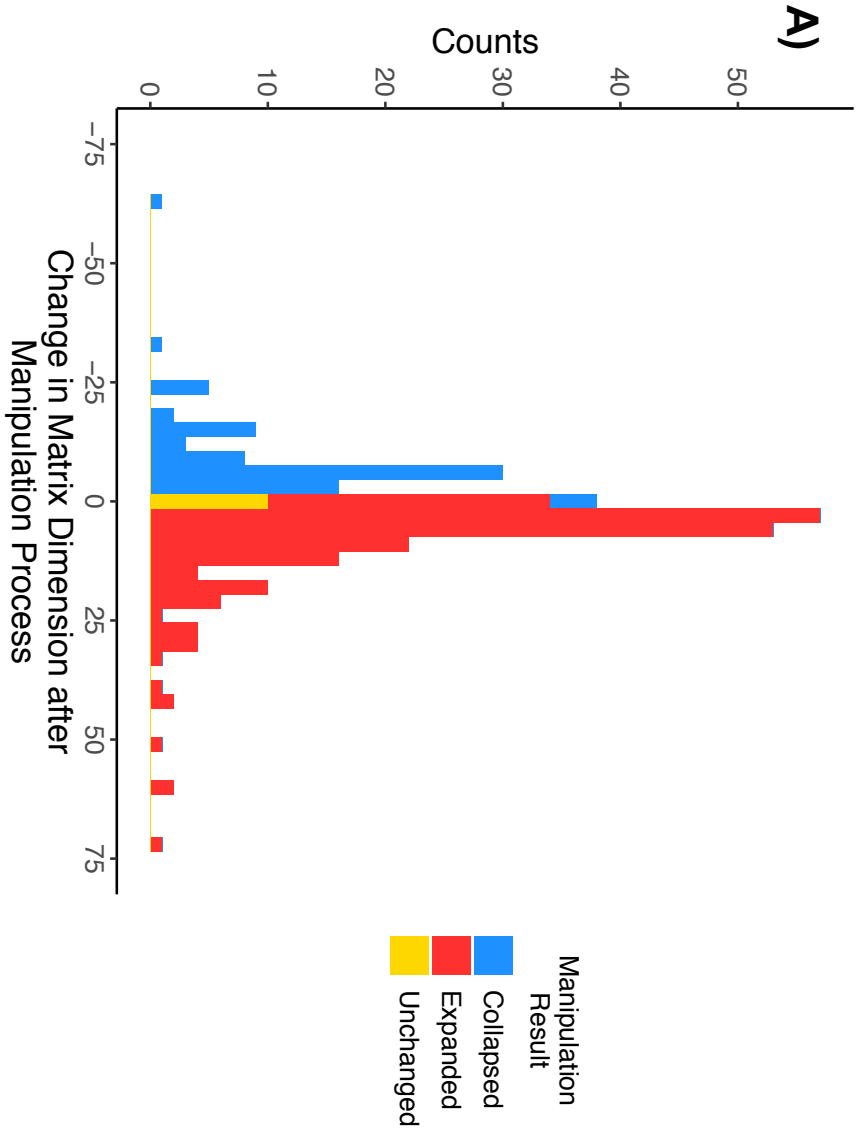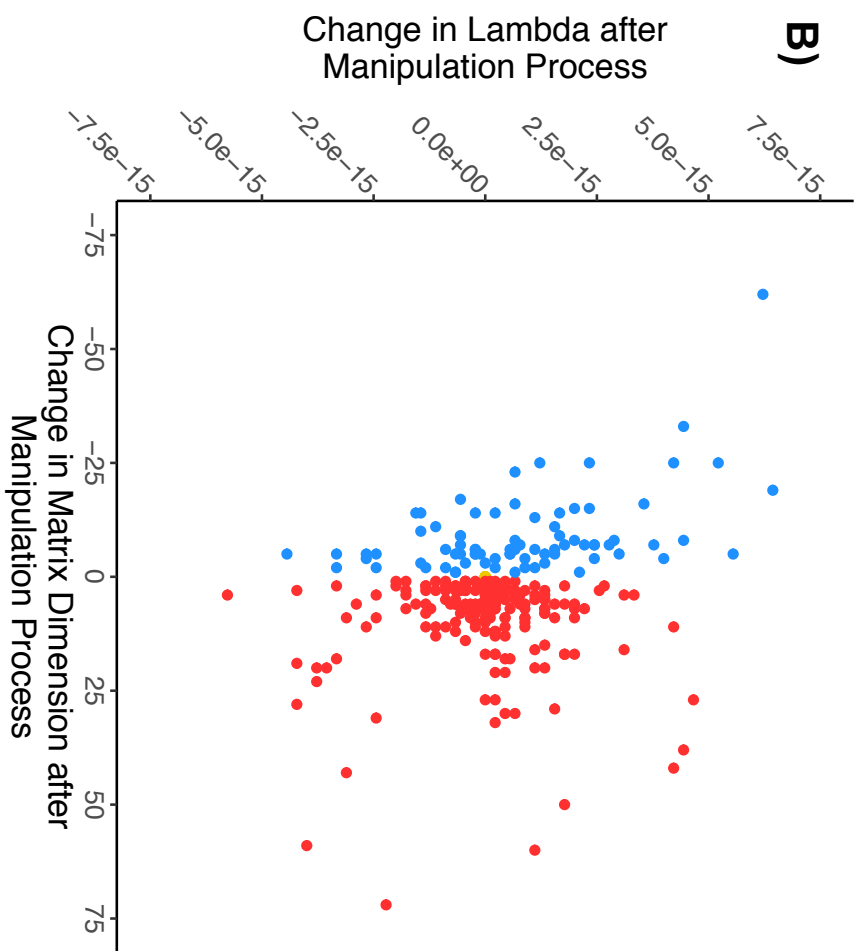

Supplement: S3 Fig — A) Histogram shows, for the 298 matrices used from the COMADRE database, how they were altered in dimension, either expanded or collapsed, and the change in dimension after alteration. B) Plot shows the change in matrix dimension and the associated difference between λ when calculated for the original matrix and when calculated for the matrix after the dimension has been altered. (PDF) [file pcbi.1009714.s003.pdf]

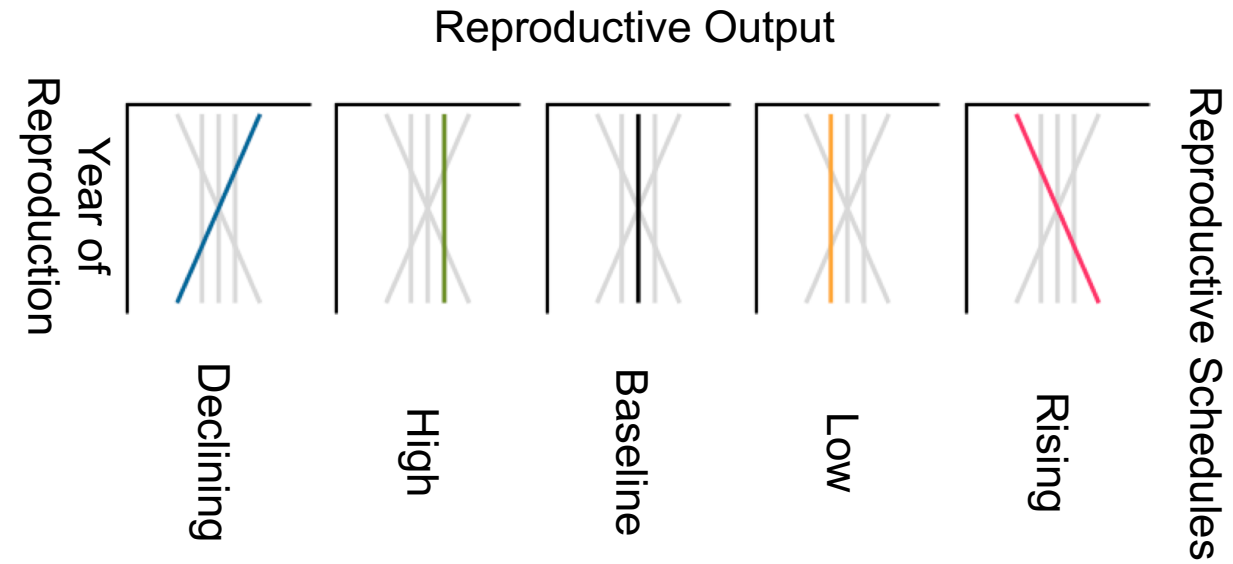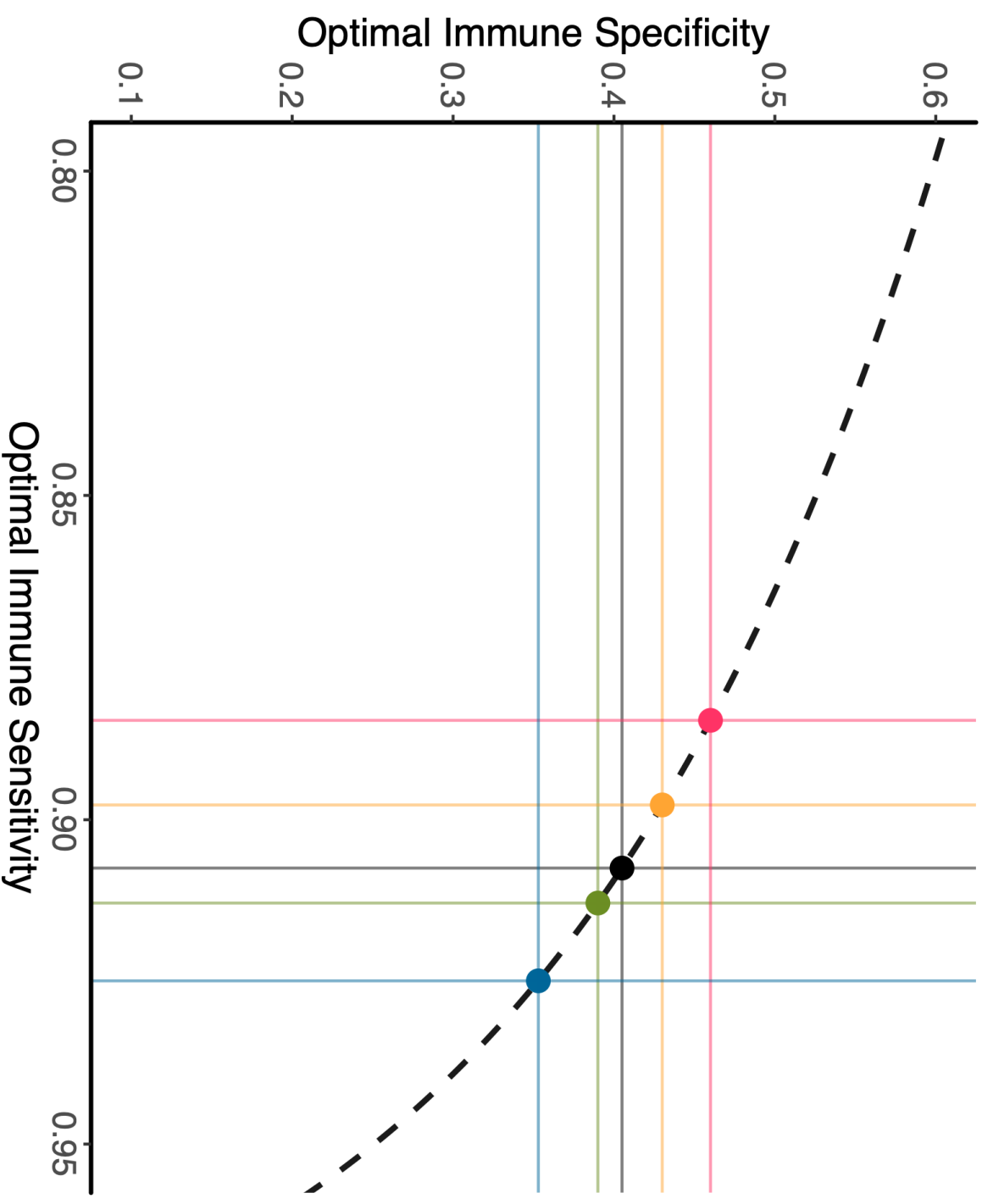

Supplement: S5 Fig — Plot showing optimal combination of immune sensitivity and specificity for each of five reproductive demographic schedules (color-coded at left), for a single epidemiological risk environment where infection risk declines at a constant rate from ir = 0.6 in age class 1 to ir = 0.2 in age class 10. Reproduction begins in the third age class for all schedules. Strategy optima, shown as points on the dashed curve, are determined as the immune specificity and sensitivity maximizing λ, the population growth rate. Dashed curve shows the shape of the specificity/sensitivity trade-off curve for γ = 4. Solid lines show values of the respective optimal strategies on each axis. Other parameter values are μb = 0.15, μi = 0.1, μd = 0.3, and μid = 0.01. (PDF) [file pcbi.1009714.s005.pdf]

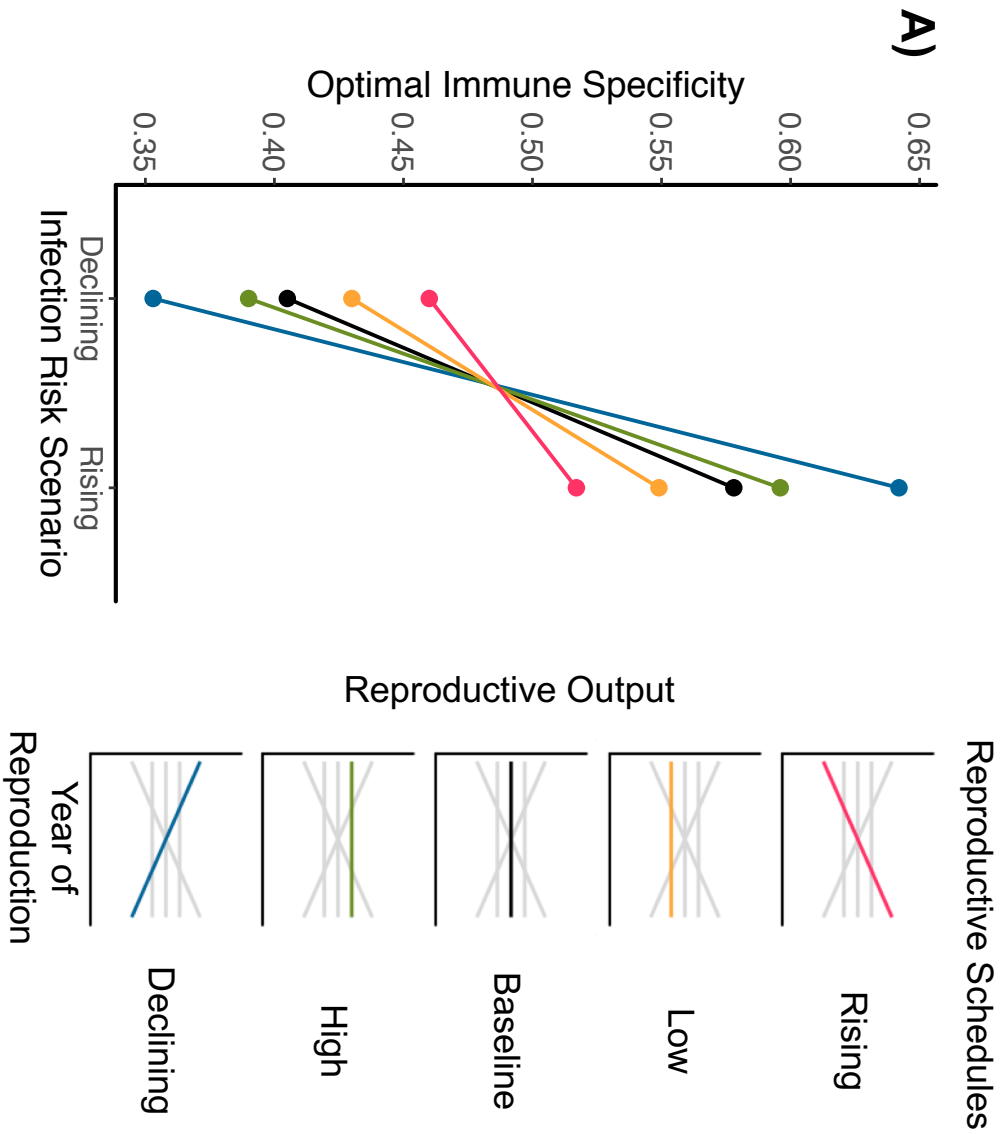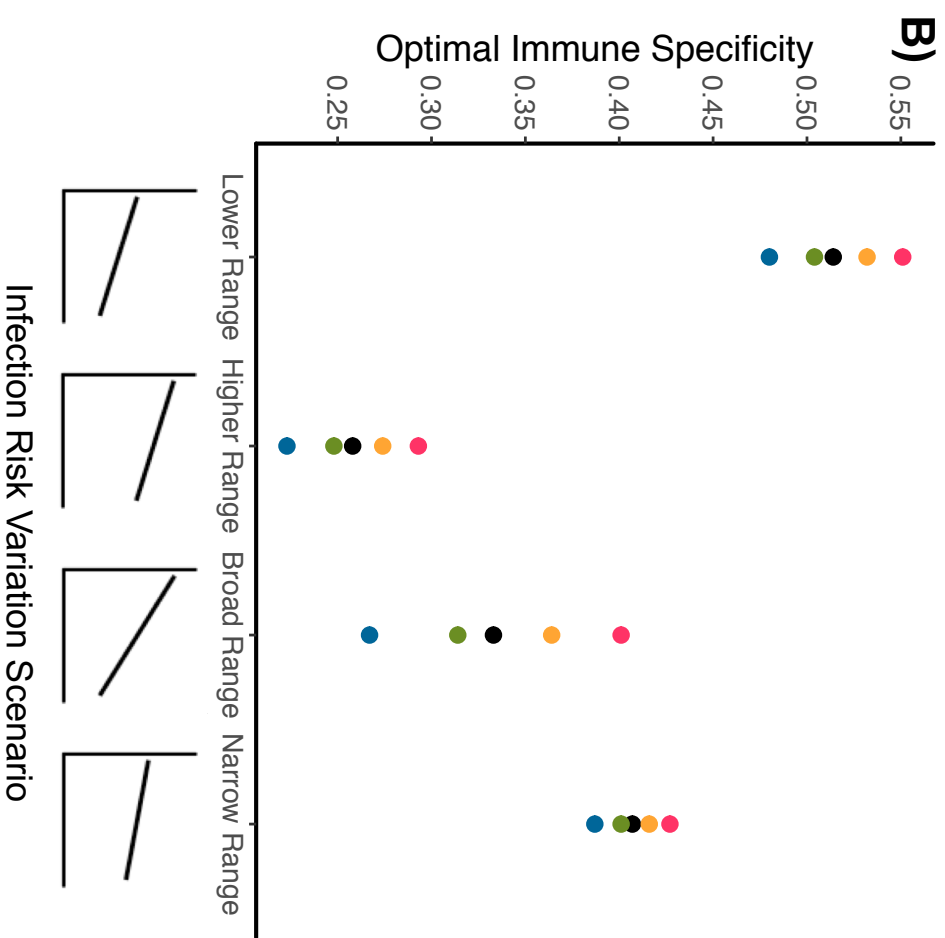

Supplement: S6 Fig — Reproduction begins in the third age class for all schedules, and ir changes a constant amount from age class to age class within each schedule. A) The change in optimal immune specificity associated with differences in epidemiological context (i.e. changes in ir, on the x-axis) and reproduction (different points and lines, color-coded at center). Parameter values are μb = 0.15, μi = 0.1, μd = 0.3, μid = 0.01, and γ = 4. In the declining scenario, ir declines with age from 0.6 to 0.2; in the rising scenario, ir increases from 0.2 to 0.6. B) The change in range of optimal specificities associated with different reproductive demographies associated with different magnitudes of variation in decline of infection risk ir with age. Parameter values are μb = 0.15, μi = 0.1, μd = 0.3, μdi = 0.01, and γ = 4. In the lower range scenario, ir declines with age from 0.45 to 0.2; in the higher range, from 0.7 to 0.45; in the broad range, from 0.7 to 0.2; in the narrow range, from 0.525 to 0.375. (PDF) [file pcbi.1009714.s006.pdf]

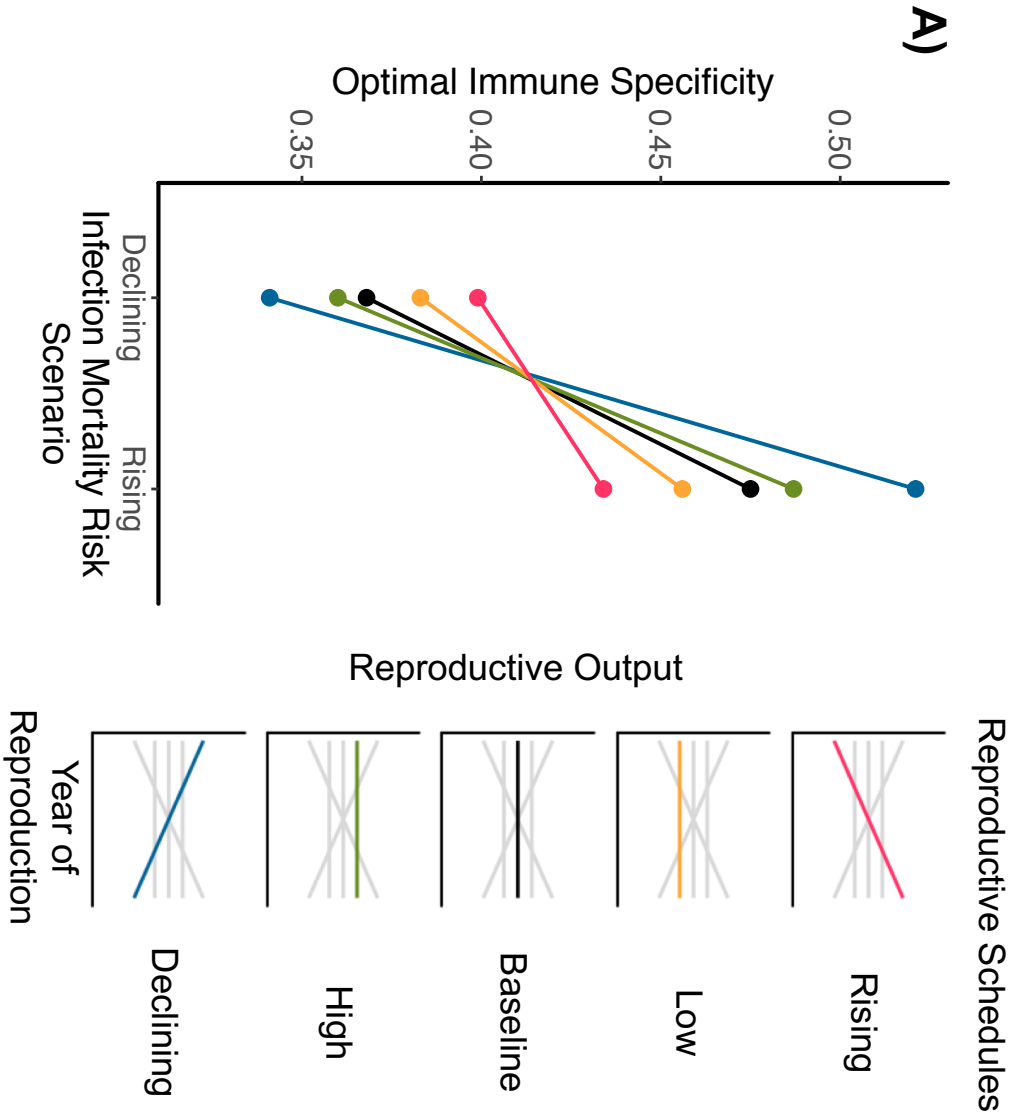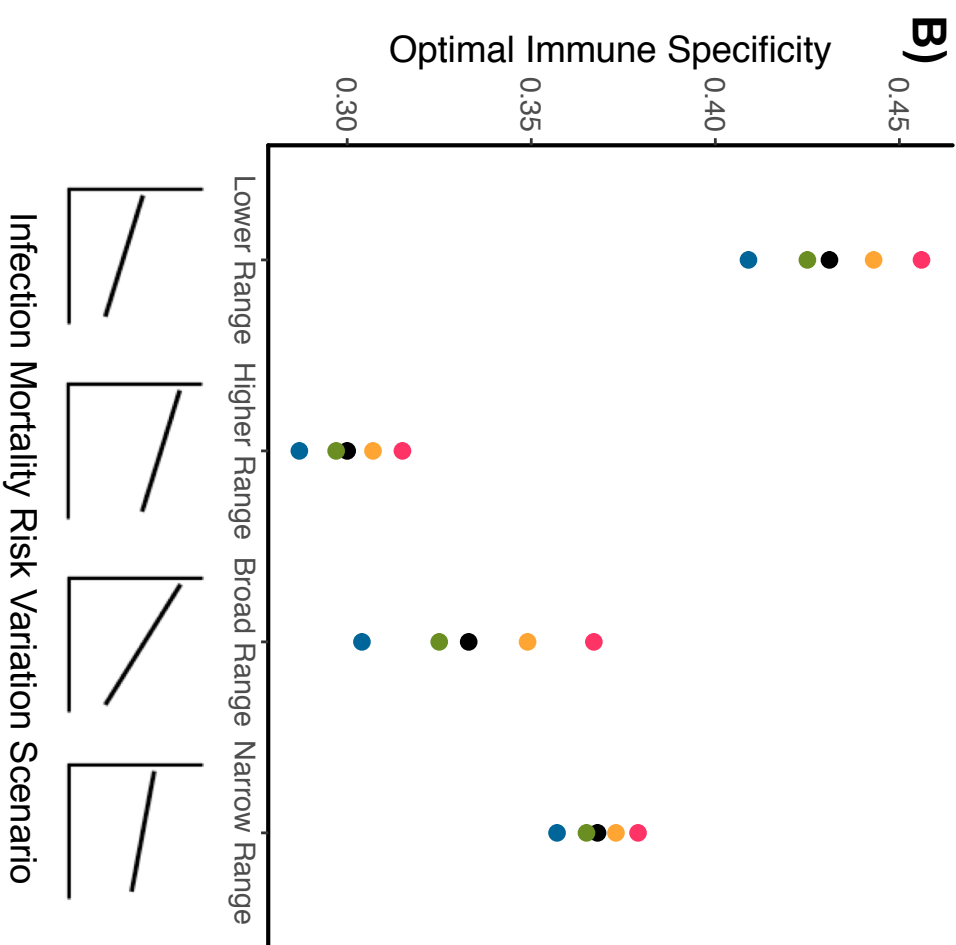

Supplement: S7 Fig — Reproduction begins in the third age class for all schedules, and μd changes a constant amount from age class to age class within each schedule. A) The change in optimal immune specificity associated with differences in epidemiological context (i.e. changes in μd, on the x-axis) and reproduction (different points and lines, color-coded at center). Parameter values are μb = 0.15, μi = 0.1, μid = 0.01, ir = 0.4, and γ = 4. In the declining scenario, μd declines with age from 0.6 to 0.2; in the rising scenario, μd increases from 0.2 to 0.6. B) The change in range of optimal specificities associated with different reproductive demographies associated with different magnitudes of variation in decline of infection mortality risk μd with age. Parameter values are μb = 0.15, μi = 0.1, μid = 0.01, ir = 0.4, and γ = 4. In the lower range scenario, μd declines with age from 0.45 to 0.2; in the higher range, from 0.7 to 0.45; in the broad range, from 0.7 to 0.2; in the narrow range, from 0.525 to 0.375. (PDF) [file pcbi.1009714.s007.pdf]

**A)**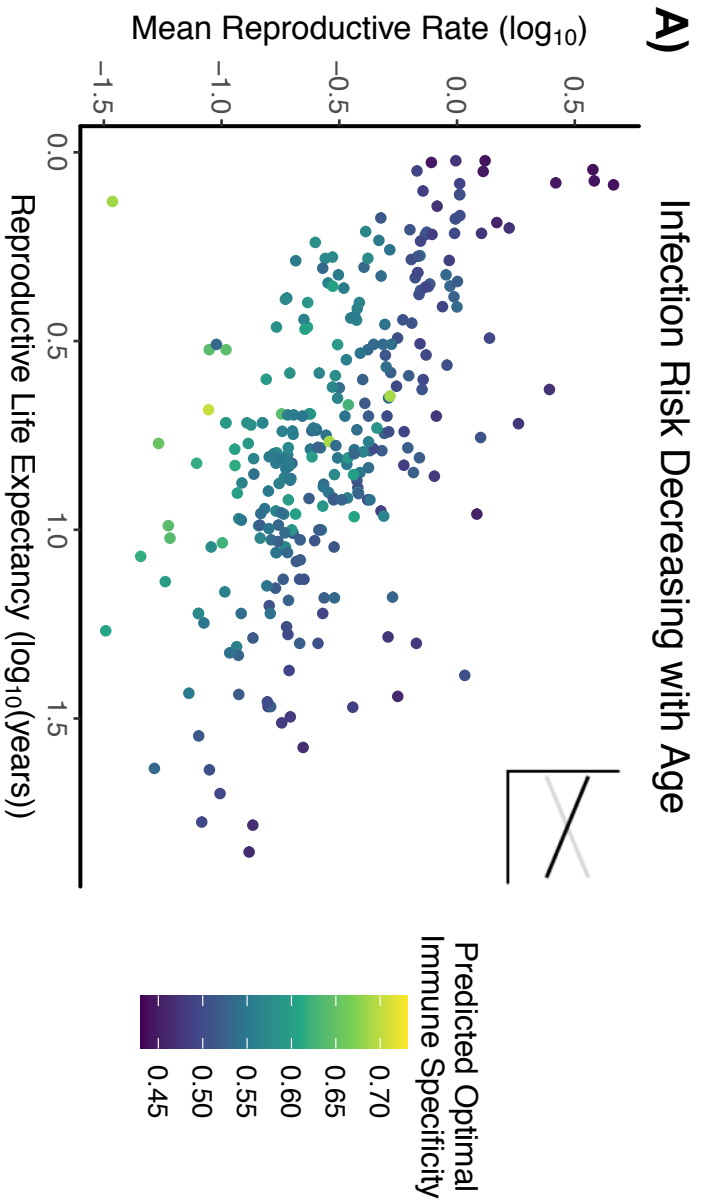**B)**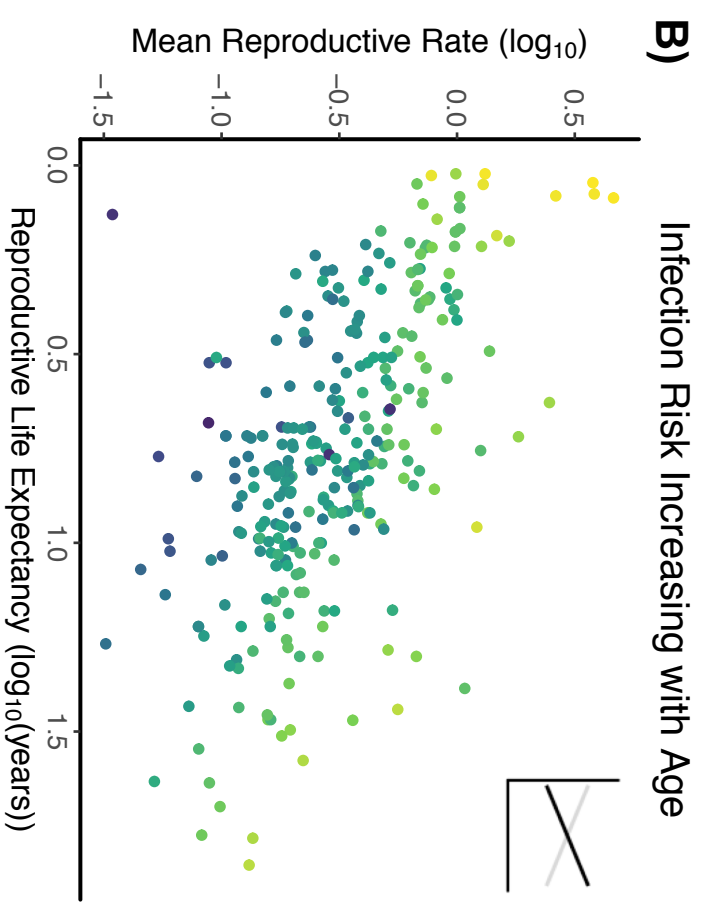

Supplement: S8 Fig — Infection risk is set such that infection risk changes at a constant rate relative to lifespan from a defined value of ir for the first age class to a defined value of ir for the last age class. Our dataset comprises 298 population matrices representing 129 chordate species. For all scenarios, parameter values are μd = 0.3, μi = 0.1, μid = 0.01, ρ = 0.75, and γ = 4. A) Predicted optimal immune specificities when infection risk declines with age, with respect to population reproductive life expectancy and mean reproductive rate as calculated from original matrix. Infection risk ir prior to reproductive maturity is 0.45; for reproductive age classes, it is 0.2. B) Predicted optimal immune specificities when infection risk rises with age with respect to population reproductive life expectancy and mean reproductive rate as calculated from original matrix. Infection risk ir prior to reproductive maturity is 0.2; for reproductive age classes, it is 0.45. (PDF) [file pcbi.1009714.s008.pdf]

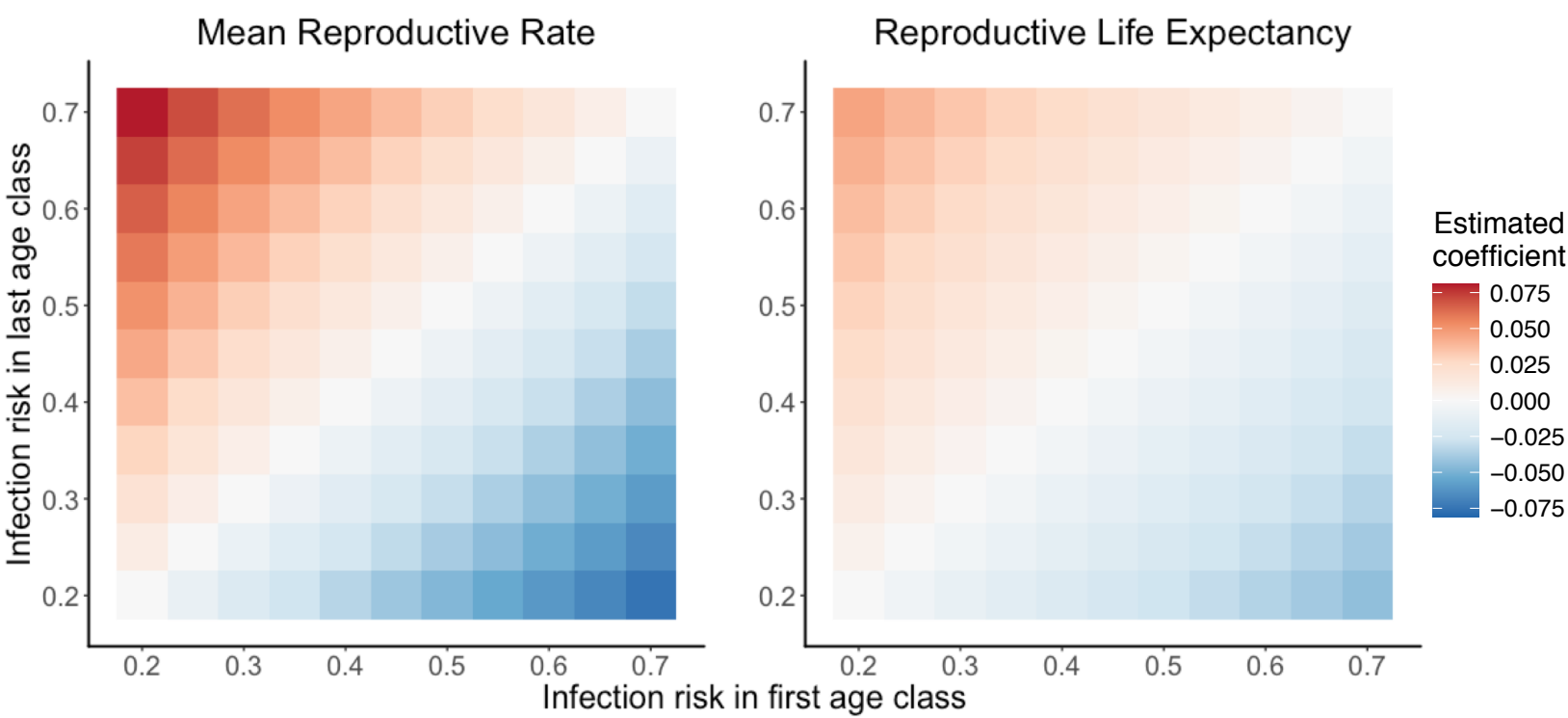

Supplement: S9 Fig — Tile plots showing, for a variety of scenarios of variation in ir, coefficients of relationship between the designated life history trait and predicted optimal immune specificity as estimated from Bayesian linear models. Each tile is a scenario in which predicted optimal immune specificities were generated for 298 population projection matrices representing 129 chordate species. A linear model was used to estimate relationship coefficients for each scenario. The coefficient value in the plot represents the mean value of the posterior probability distribution, except on the diagonal; on the diagonal, all coefficients are 0 (see text). The value of ir on the x-axis is the risk for the first age class in the matrix, while the value on the y-axis is the risk for the last age class; rate of change between intermediate age classes is adjusted per matrix, based on dimension, so that risk changes at a constant rate from age class to age class within a matrix but the absolute magnitude of risk change is equivalent for all matrices. Life history trait values log-transformed and standardized as Z-scores for comparability of coefficients. For all estimated coefficients off the diagonal, 89% credible intervals do not include 0. For all scenarios, parameter values are μd = 0.3, μi = 0.1, μid = 0.01, ρ = 0.75, and γ = 4. Unlike Fig 5, age class of first reproduction is not shown because our models do not confidently predict a relationship with immune specificity for any ir scenario. (PDF) [file pcbi.1009714.s009.pdf]
