# Supplementary material for: Optimal immune specificity at the intersection of host life history and parasite epidemiology
Source: PLoS Comput Biol. 2021 Dec 21;17(12):e1009714. doi: 10.1371/journal.pcbi.1009714 (PMC8730424; doi:10.1371/journal.pcbi.1009714)
Supplement: S4 Fig — X-coordinates for lines in absolute age plots determined by age classes within matrix, and so the blue line is for an organism with a short lifespan and smaller matrix dimension, while the red line is for an organism with a long lifespan and larger matrix dimension. Dimension of manipulated matrices (see S3 Fig) determined as sum of age at first reproduction and reproductive life expectancy. (PDF) [file pcbi.1009714.s004.pdf]

How to scale infection risk i_r schedules for different lifespans?

1) Identify i_r values for first and last age class
2) Determine lifespan of organism, expressed as the number of age classes in matrix
3) Scale rate of change in i_r between age classes by total number of age classes

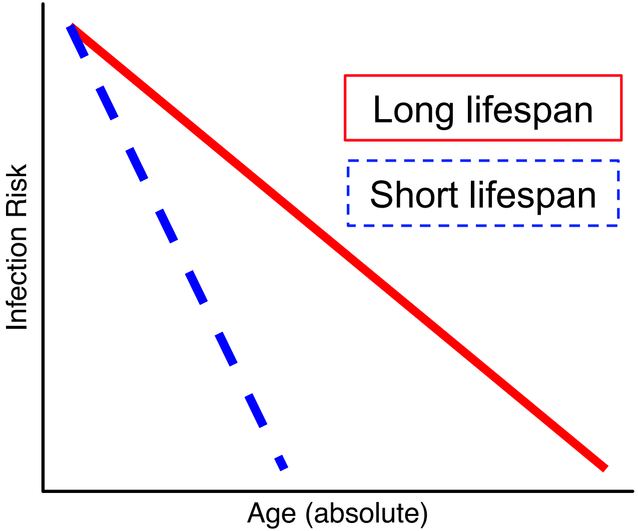

Different rates of absolute i_r change, but…

= (with scaling)

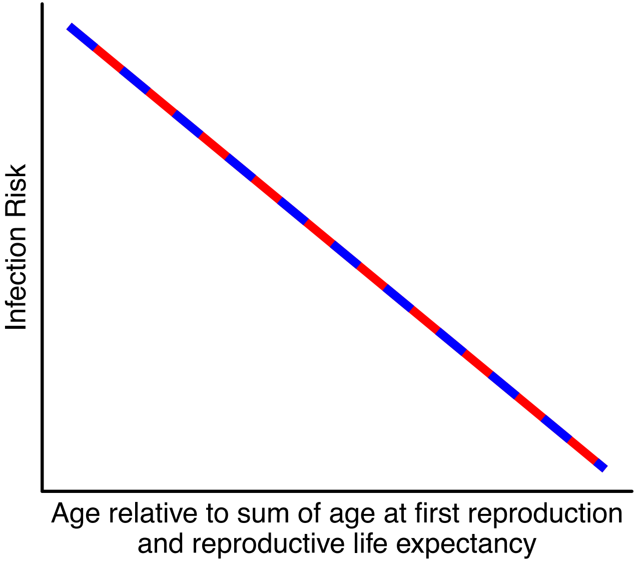

…the same rate of relative i_r change.
