# Supplementary material for: Optimal immune specificity at the intersection of host life history and parasite epidemiology
Source: PLoS Comput Biol. 2021 Dec 21;17(12):e1009714. doi: 10.1371/journal.pcbi.1009714 (PMC8730424; doi:10.1371/journal.pcbi.1009714)
Supplement: S1 Table — Comparison of results from analysis of effect of reproductive schedule on optimal immune strategy when μb does not change to equalize λ (original) and when μb does change to equalize λ (adjusted). sp* is the optimal immune specificity that maximizes λ. Infection risk ir declines from 0.6 before reproductive maturity (age classes 1 and 2) to 0.2 after (classes 3+). Other parameter values are μi = 0.1, μd = 0.3, μid = 0.01, and γ = 4. (DOCX) [file pcbi.1009714.s010.docx]

**S1 Table. Effect of background mortality *µ_b_* on optimal immune strategy.** Comparison of results from analysis of effect of reproductive schedule on optimal immune strategy when *µ_b_* does not change to equalize λ (original) and when *µ_b_* does change to equalize λ (adjusted). *s_p_^*^* is the optimal immune specificity that maximizes λ. Infection risk *i_r_* declines from 0.6 before reproductive maturity (age classes 1 and 2) to 0.2 after (classes 3+). Other parameter values are *µ_i_* = 0.1, *µ_d_* = 0.3, *µ_id_* = 0.01, and γ = 4.

| Reproductive Schedule | Original *µ_b_* | Original λ_max_ | Original *s_p_^*^* | Adjusted *µ_b_* | Adjusted λ_max_ | Adjusted *s_p_^*^* |
| --- | --- | --- | --- | --- | --- | --- |
| Rising | 0.150 | 1.089 | 0.590 | 0.105 | 1.133 | 0.591 |
| Low | 0.150 | 1.059 | 0.545 | 0.070 | 1.133 | 0.551 |
| Baseline | 0.150 | 1.133 | 0.517 | 0.150 | 1.133 | 0.517 |
| High | 0.150 | 1.193 | 0.497 | 0.216 | 1.133 | 0.493 |
| Declining | 0.150 | 1.199 | 0.439 | 0.224 | 1.133 | 0.436 |
