# Supplementary material for: Optimal immune specificity at the intersection of host life history and parasite epidemiology
Source: PLoS Comput Biol. 2021 Dec 21;17(12):e1009714. doi: 10.1371/journal.pcbi.1009714 (PMC8730424; doi:10.1371/journal.pcbi.1009714)
Supplement: S2 Table — Comparison of results from analysis of reproductive schedule on optimal immune strategy when μb does not change to equalize λ (original) and when μb does change to equalize λ (adjusted). sp* is the optimal immune specificity that maximizes λ. Infection risk ir declines smoothly from 0.6 in the first age class to 0.2 in the final age class. Other parameter values are μi = 0.1, μd = 0.3, μid = 0.01, and γ = 4. (DOCX) [file pcbi.1009714.s011.docx]

**S2 Table. Effect of background mortality *µ_b_* on optimal immune strategy: smoothed infection risk variation.** Comparison of results from analysis of reproductive schedule on optimal immune strategy when *µ_b_* does not change to equalize λ (original) and when *µ_b_* does change to equalize λ (adjusted). *s_p_^*^* is the optimal immune specificity that maximizes λ. Infection risk *i_r_* declines smoothly from 0.6 in the first age class to 0.2 in the final age class. Other parameter values are *µ_i_* = 0.1, *µ_d_* = 0.3, *µ_id_* = 0.01, and γ = 4.

| Reproductive Schedule | Original *µ_b_* | Original λ_max_ | Original *s_p_^*^* | Adjusted *µ_b_* | Adjusted λ_max_ | Adjusted *s_p_^*^* |
| --- | --- | --- | --- | --- | --- | --- |
| Rising | 0.150 | 1.088 | 0.460 | 0.104 | 1.133 | 0.462 |
| Low | 0.150 | 1.059 | 0.430 | 0.070 | 1.133 | 0.436 |
| Baseline | 0.150 | 1.134 | 0.405 | 0.150 | 1.134 | 0.405 |
| High | 0.150 | 1.196 | 0.390 | 0.217 | 1.133 | 0.387 |
| Declining | 0.150 | 1.201 | 0.353 | 0.226 | 1.133 | 0.351 |
