# Supplementary material for: Optimal immune specificity at the intersection of host life history and parasite epidemiology
Source: PLoS Comput Biol. 2021 Dec 21;17(12):e1009714. doi: 10.1371/journal.pcbi.1009714 (PMC8730424; doi:10.1371/journal.pcbi.1009714)
Supplement: S3 Table — Linear model looks at model-predicted optimal immune specificity as a function of three different life history summary statistics. Results are means and, in brackets, boundaries of 89% highest posterior density intervals (HPDI) for posterior probability distributions for parameter values. Entries in italics indicate the 89% HPDI overlaps with 0 for that parameter. All summary statistics were calculated from original matrix in COMADRE database, log-transformed, and standardized as Z-scores. The only matrices included are those 151 qualifying matrices from 47 different mammal species. For stepped epidemiological scenario, when infection risk (ir) is rising, ir in pre-reproductive years is 0.2, and ir in reproductive years is 0.45. When infection risk declines in the stepped scenario, ir in pre-reproductive years is 0.45, and ir in reproductive years is 0.2. In smoothed declining scenario, ir declines from 0.45 to 0.2; in rising scenario, ir rises from 0.2 to 0.45. Other parameter values are μd = 0.3, μi = 0.1, μid = 0.01, ρ = 0.75, and γ = 4. (DOCX) [file pcbi.1009714.s012.docx]

**S3 Table. Results from Bayesian linear model for demography and immune strategy – analysis using only mammal life histories.** Linear model looks at model-predicted optimal immune specificity as a function of three different life history summary statistics. Results are means and, in brackets, boundaries of 89% highest posterior density intervals (HPDI) for posterior probability distributions for parameter values. Entries in *italics* indicate the 89% HPDI overlaps with 0 for that parameter. All variables calculated from original matrix in COMADRE database, log-transformed, and standardized as Z-scores. The only matrices included are those 151 qualifying matrices from 47 different mammal species. For stepped epidemiological scenario, when infection risk (*i_r_*) is rising, *i_r_* in pre-reproductive years is 0.2, and *i_r_* in reproductive years is 0.45. When infection risk declines in the stepped scenario, *i_r_* in pre-reproductive years is 0.45, and *i_r_* in reproductive years is 0.2. In smoothed declining scenario, *i_r_* declines from 0.45 to 0.2; in rising scenario, *i_r_* rises from 0.2 to 0.45. Other parameter values are *µ_d_* = 0.3, *µ_i_* = 0.1, *µ_id_* = 0.01, ρ = 0.75, and γ = 4.

| Parameter | Declining stepped infection risk *i_r_* | Rising stepped infection risk *i_r_* | Declining smoothed infection risk *i_r_* | Rising smoothed infection risk *i_r_* |
| --- | --- | --- | --- | --- |
| Intercept | 0.626  [0.624, 0.629] | 0.521  [0.518, 0.523] | 0.543  [0.540, 0.546] | 0.603  [0.599, 0.606] |
| Age class of first reproduction | -0.0407  [-0.0442, -0.0373] | 0.0356  [0.0326, 0.0384] | -0.00426  [-0.00830, -3.74x10^-4^] | *0.00311*  *[-8.69x10^-4^, 0.00699]* |
| Mean reproductive rate | -0.0309  [-0.0341, -0.0275] | 0.0266  [0.0239, 0.0293] | -0.0325  [-0.0363, -0.0285] | 0.0330  [0.0291, 0.0369] |
| Reproductive life expectancy | 0.00988  [0.00675, 0.0130] | -0.00894  [-0.0110, -0.00578] | -0.0313  [-0.0349, -0.0275] | 0.0342  [0.0306, 0.0379] |
| Standard deviation | 0.0202  [0.0184, 0.0222] | 0.0176  [0.0160, 0.0194] | 0.0239  [0.0216, 0.0262] | 0.0247  [0.0224, 0.0271] |
