# Supplementary material for: Optimal immune specificity at the intersection of host life history and parasite epidemiology
Source: PLoS Comput Biol. 2021 Dec 21;17(12):e1009714. doi: 10.1371/journal.pcbi.1009714 (PMC8730424; doi:10.1371/journal.pcbi.1009714)
Supplement: S4 Table — Linear model looks at model-predicted optimal immune specificity as a function of the common life history summary statistic generation time, which is heavily confounded with other summary statistics. Results are means and, in brackets, boundaries of 89% highest posterior density intervals. All summary statistics were calculated from original matrix in COMADRE database, log-transformed, and standardized as Z-scores. Dataset includes 298 qualifying matrices from 129 chordate species. In smoothed declining scenario, infection risk (ir) declines from 0.45 to 0.2; in rising scenario, ir rises from 0.2 to 0.45. For stepped epidemiological scenario, when infection risk is rising, ir in pre-reproductive years is 0.2, and ir in reproductive years is 0.45. When infection risk declines in the stepped scenario, ir in pre-reproductive years is 0.45, and ir in reproductive years is 0.2. In smoothed declining scenario, ir declines from 0.45 to 0.2; in rising scenario, ir rises from 0.2 to 0.45. Other parameter values are μd = 0.3, μi = 0.1, μid = 0.01, ρ = 0.75, and γ = 4. (DOCX) [file pcbi.1009714.s013.docx]

**S4 Table. Results from Bayesian linear model for demography and immune strategy – analysis with generation time only.** Linear model looks at model-predicted optimal immune specificity as a function of the common life history summary statistic generation time, which is heavily confounded with other summary statistics. Results are means and, in brackets, boundaries of 89% highest posterior density intervals. All variables calculated from original matrix in COMADRE database, log-transformed, and standardized as Z-scores. Dataset includes 298 qualifying matrices from 129 chordate species. In smoothed declining scenario, infection risk (*i_r_*) declines from 0.45 to 0.2; in rising scenario, *i_r_* rises from 0.2 to 0.45. For stepped epidemiological scenario, when infection risk is rising, *i_r_* in pre-reproductive years is 0.2, and *i_r_* in reproductive years is 0.45. When infection risk declines in the stepped scenario, *i_r_* in pre-reproductive years is 0.45, and *i_r_* in reproductive years is 0.2. In smoothed declining scenario, *i_r_* declines from 0.45 to 0.2; in rising scenario, *i_r_* rises from 0.2 to 0.45. Other parameter values are *µ_d_* = 0.3, *µ_i_* = 0.1, *µ_id_* = 0.01, ρ = 0.75, and γ = 4.

| Parameter | Declining stepped infection risk *i_r_* | Rising stepped infection risk *i_r_* | Declining smoothed infection risk *i_r_* | Rising smoothed infection risk *i_r_* |
| --- | --- | --- | --- | --- |
| Intercept | 0.603  [0.598, 0.608] | 0.544  [0.539, 0.548] | 0.538  [0.534, 0.542] | 0.608  [0.604, 0.611] |
| Generation time | 0.0268  [0.0221, 0.0315] | -0.0262  [-0.0308, -0.0216] | 0.0156  [0.0119, 0.0196] | -0.0168  [-0.0212, -0.0123] |
| Standard deviation | 0.0519  [0.0485, 0.0557] | 0.0503  [0.0471, 0.0540] | 0.0418  [0.0393, 0.0446] | 0.447  [0.0419, 0.0478] |
