# Supplementary material for: Optimal immune specificity at the intersection of host life history and parasite epidemiology
Source: PLoS Comput Biol. 2021 Dec 21;17(12):e1009714. doi: 10.1371/journal.pcbi.1009714 (PMC8730424; doi:10.1371/journal.pcbi.1009714)
Supplement: S5 Table — Linear model looks at model-predicted optimal immune specificity as a function of three different life history summary statistics. Results are means and, in brackets, boundaries of 89% highest posterior density intervals (HPDI) for posterior probability distributions for parameter values. Entries in italics indicate the 89% HPDI overlaps with 0 for that parameter. All summary statistics were calculated from output matrices after manipulation and optimization of sp (unlike results shown in Fig 4 and Table 1), log-transformed, and standardized as Z-scores. Dataset includes 298 qualifying matrices from 129 chordate species. For stepped epidemiological scenario, when infection risk (ir) is declining, ir in pre-reproductive years is 0.2, and ir in reproductive years is 0.45. When infection risk declines in the stepped scenario, ir in pre-reproductive years is 0.45, and ir in reproductive years is 0.2. In smoothed declining scenario, ir declines from 0.45 to 0.2; in rising scenario, ir rises from 0.2 to 0.45. Other parameter values are μd = 0.3, μi = 0.1, μid = 0.01, ρ = 0.75, and γ = 4. (DOCX) [file pcbi.1009714.s014.docx]

**S5 Table. Results from Bayesian linear model for demography and immune strategy – analysis with post-processing life history statistics.** Linear model looks at model-predicted optimal immune specificity as a function of three different life history summary statistics. Results are means and, in brackets, boundaries of 89% highest posterior density intervals (HPDI) for posterior probability distributions for parameter values. Entries in *italics* indicate the 89% HPDI overlaps with 0 for that parameter. All variables calculated from output matrices after manipulation and optimization of *s_p_* (unlike results shown in Fig. 3 and Table 1), log-transformed, and standardized as Z-scores. Dataset includes 298 qualifying matrices from 129 chordate species. For stepped epidemiological scenario, when infection risk (*i_r_*) is declining, *i_r_* in pre-reproductive years is 0.2, and *i_r_* in reproductive years is 0.45. When infection risk declines in the stepped scenario, *i_r_* in pre-reproductive years is 0.45, and *i_r_* in reproductive years is 0.2. In smoothed declining scenario, *i_r_* declines from 0.45 to 0.2; in rising scenario, *i_r_* rises from 0.2 to 0.45. Other parameter values are *µ_d_* = 0.3, *µ_i_* = 0.1, *µ_id_* = 0.01, ρ = 0.75, and γ = 4.

| Parameter | Declining stepped infection risk *i_r_* | Rising stepped infection risk *i_r_* | Declining smoothed infection risk *i_r_* | Rising smoothed infection risk *i_r_* |
| --- | --- | --- | --- | --- |
| Intercept | 0.603  [0.600, 0.605] | 0.544  [0.541, 0.547] | 0.538  [0.536, 0.541] | 0.608  [0.605, 0.611] |
| Age class of first reproduction | -0.0379  [-0.0406, -0.0352] | 0.0359  [0.0333, 0.0386] | *2.60x10^-4^*  *[-0.00304, 0.00351]* | *-5.27x10^-4^*  *[-0.00387, 0.00300]* |
| Mean reproductive rate | -0.0420  [-0.0453, -0.0386] | 0.0407  [0.0374, 0.0439] | 0.0415  [-0.0452, -0.0376] | 0.0443  [0.0403, 0.0484] |
| Reproductive life expectancy | 0.0190  [0.0156, 0.0226] | -0.0193  [-0.0228, -0.0160] | -0.0223  [-0.0263, -0.0183] | 0.0236  [0.0194, 0.0277] |
| Standard deviation | 0.0266  [0.0249, 0.0283] | 0.0261  [0.0243, 0.0279] | 0.0314  [0.0294, 0.0334] | 0.0334  [0.0313, 0.0356] |
