# Supplementary material for: Optimal immune specificity at the intersection of host life history and parasite epidemiology
Source: PLoS Comput Biol. 2021 Dec 21;17(12):e1009714. doi: 10.1371/journal.pcbi.1009714 (PMC8730424; doi:10.1371/journal.pcbi.1009714)
Supplement: S6 Table — The parameter values for each different analysis of the reproductive demography-immune strategy relationship as described in the Methods. We also considered similar scenarios in which infection mortality risk (μd) varies on the same intervals as infection risk (ir) does here, while ir is held constant at 0.4. For Figs 3 and S2, risk parameters changed from the first value in brackets for pre-reproductive age classes (classes 1 and 2) to the second for reproductive age classes (classes 3+). For S5 and S6 Figs, infection risk smoothly changed from the first value in the brackets in the first age class to the second value in the last age class of the matrix, with the same change in risk between each age class. (DOCX) [file pcbi.1009714.s015.docx]

**S6 Table. Epidemiological Risk Scenarios: Set A.** The parameter values for each different analysis of the reproductive demography-immune strategy relationship as described in the Methods. We also considered similar scenarios in which infection mortality risk (*µ_d_*) varies on the same intervals as *i_r_* does here, while infection risk (*i_r_*) is held constant at 0.4. For Figures 3 and S4, risk parameters changed from the first value in brackets for pre-reproductive age classes (classes 1 and 2) to the second for reproductive age classes (classes 3+). For Figures S5 and S6, infection risk smoothly changed from the first value in the brackets in the first age class to the second value in the last age class of the matrix, with the same change in risk between each age class.

| Parameter | Scenario A1:  Rising risk | Scenario A2:  Declining risk |
| --- | --- | --- |
| *i_r_* | [0.6, 0.2] | [0.2, 0.6] |
| *µ_b_* | 0.15 (identical for all scenarios) | |
| *µ_i_* | 0.1 (identical for all scenarios) | |
| *µ_d_* | 0.3 | 0.3 |
| *µ_id_* | 0.01 (identical for all scenarios) | |
| *γ* | 4 (identical for all scenarios) | |
