# Supplementary material for: Optimal immune specificity at the intersection of host life history and parasite epidemiology
Source: PLoS Comput Biol. 2021 Dec 21;17(12):e1009714. doi: 10.1371/journal.pcbi.1009714 (PMC8730424; doi:10.1371/journal.pcbi.1009714)
Supplement: S7 Table — The parameter values for each scenario of the reproductive demography-immune strategy relationship as described in the Methods, used for an analysis of the influence of magnitude of change in risk with age on immune strategy. We also considered scenarios where infection mortality risk (μd) varied on the same intervals as infection risk (ir) does here, with ir then being held at 0.4 across all age classes. For Figs 3 and S2, risk parameters dropped from the first value in brackets for pre-reproductive age classes (classes 1 and 2) to the second for reproductive age classes (classes 3+). For S5 and S6 Figs, infection risk smoothly fell from the first value in the brackets in the first age class to the second value in the last age class of the matrix, with the same change in risk between each age class. (DOCX) [file pcbi.1009714.s016.docx]

**S7 Table. Epidemiological Risk Scenarios: Set B.** The parameter values for each scenario of the reproductive demography-immune strategy relationship as described in the Methods, used for an analysis of the influence of magnitude of change in risk with age on immune strategy. We also considered scenarios where infection mortality risk (*µ_d_*) varied on the same intervals as infection risk (*i_r_*) does here, with *i_r_* then being held at 0.4 across all age classes. For Figures 3 and S4, risk parameters dropped from the first value in brackets for pre-reproductive age classes (classes 1 and 2) to the second for reproductive age classes (classes 3+). For Figures S5 and S6, infection risk smoothly fell from the first value in the brackets in the first age class to the second value in the last age class of the matrix, with the same change in risk between each age class.

| Parameter | Scenario B1: Low range | Scenario B2: High range | | Scenario B3: Broad range | | Scenario B4: Narrow range | |
| --- | --- | --- | --- | --- | --- | --- | --- |
| *i_r_* | [0.45, 0.2] | [0.7, 0.45] | | [0.7, 0.2] | | [0.525, 0.375] | |
| *µ_b_* | 0.15 (identical for all scenarios) | | | | | | |
| *µ_i_* | 0.1 (identical for all scenarios) | | | | | | |
| *µ_d_* | 0.3 | | 0.3 | | 0.3 | | 0.3 |
| *µ_id_* | 0.01 (identical for all scenarios) | | | | | | |
| *γ* | 4 (identical for all scenarios) | | | | | | |
