# Supplementary material for: Optimal immune specificity at the intersection of host life history and parasite epidemiology
Source: PLoS Comput Biol. 2021 Dec 21;17(12):e1009714. doi: 10.1371/journal.pcbi.1009714 (PMC8730424; doi:10.1371/journal.pcbi.1009714)
Supplement: Attachment — Submitted filename: Downieetal.2021.reviewer.responses.docx [file pcbi.1009714.s018.docx]

*[1] A letter containing a detailed list of your responses to all review comments, and a description of the changes you have made in the manuscript. Please note while forming your response, if your article is accepted, you may have the opportunity to make the peer review history publicly available. The record will include editor decision letters (with reviews) and your responses to reviewer comments. If eligible, we will contact you to opt in or out*

*[2] Two versions of the revised manuscript: one with either highlights or tracked changes denoting where the text has been changed; the other a clean version (uploaded as the manuscript file).*

Dear Editors:

Thank you very much for your response to our manuscript and for the opportunity to revise it. We detail our responses to the reviewers’ comments below; their comments are italicized, while our responses are in standard font. We have tried to balance suggestions for places to trim the text (mostly from Reviewer 1) with suggestions for places to delve into a little more detail (mostly from Reviewers 2 and 3) so as not to lengthen the text too much more. In addition to the textual changes, we have made edits to Figures 2, S2, and S5 as requested by our reviewers, and have made a very minor corresponding change to our submitted code for generating Figures 2 and S5.

We hope that our revisions are satisfactory and very much look forward to hearing back from you soon.

Best wishes,

Alexander E. Downie

Andreas Mayer

C. Jessica E. Metcalf

Andrea L. Graham

Reviewer Comments and Responses

*Reviewer #1: In the manuscript “Optimal immune specificity at the intersection of host life history and parasite epidemiology”, the authors applied their previously-developed model to investigate the optimal immune strategies in terms of immune specificity by varying the reproduction schedules and key parameters such as the infection risk i_r. Further incorporating data from COMADRE Animal Matrix Database, the authors explored the association between the optimal immune strategies and three life history traits, suggesting that both demographic and epidemiological factors can greatly alter the optimal immune strategies. Overall, this work is solid and provides some interesting and useful insights into how the immune strategies change against epidemiological and demographic variations.*

We thank the reviewer for their positive feedback and interest.

*On the other hand, I feel that the presentation could be improved to better explain how the computational model is implemented – the high-level description of matrix computation is not very clear, as it is very important in the modelling part. While most of the significant results depends on the previously-developed model and the associated model assumptions, the significance of computational work needs to be explicitly emphasized.*

We agree that the description can be made clearer, especially in the Introduction and Results sections, and we have accordingly added some explanation of how the survival and fertility parameters are defined for each of these sections. We have also revised the descriptions in the Methods to make them clearer and more useful as a reference for understanding the details of our computational approach.

*Also the Discussion section is unnecessarily long and therefore loses its focus. Therefore, I suggest the authors revising the manuscript so that readers can better link the results to the model set-up, and therefore better understand the interpretation of the results.*

We agree that the Discussion would benefit from greater structure and trimming. We have accordingly done some editing throughout to make it shorter and clearer. Where we have extraneous points (such as the “for example” example highlighted by the Reviewer below), we have deleted unnecessary text. We have also added headings within the Discussion section that will signpost the reader, to help with organization and allow them to focus on points that are relevant to their particular interests. We think that these changes have brought clearer focus to the Discussion.

We are reluctant to shrink too dramatically the discussion of methods and applications of our work in disease ecology, because when we have presented versions of this work at conferences we almost invariably get the question “How would you test this?” We therefore think it is important that we provide some details on this front, as well as particular empirical contexts where our framework of sensitivity and specificity could be especially relevant. Other reviewers have also requested a few additional topics for exploration in our Discussion; we have endeavored to keep those additions brief and within focus.

*Below are some further comments:

1. Figure 1 (C) I looks like a histogram instead of a curve to me. And it is not very clear how (B) and (C) are connected – how the immune response threshold in (C) is reflected in (B)?*

We very much understand the confusion – another reviewer pointed out that we had inadvertently swapped the captions for B) and C) in this figure! We have now flipped them back to their proper positions, and this should clear up the issue.

*2. Line 179 – What is “elasticity of lambda”? How is it defined? This quantity also appears in Figure S3 (C) and (D) and may need an explicit definition.*

Thank you very much for drawing this oversight to our attention. We have added a definition here, as well as some text in the Discussion where it is next mentioned to remind the reader of what elasticity is and why it is helpful for understanding our results.

*3. Age class is intensely discussed in the Results section is the key forming matrices for computation – what do they represent in different subsections? In all the cases studied, do they have the same structure of age classes? When incorporating the data, it seems that the age class is on a yearly basis, is it the same for the other computational cases?*

We appreciate the suggestion and have added a little more text in the Introduction and Results sections highlighting that all age classes are of the same length and that, in the case of the COMADRE database, that length is one year. For the non-COMADRE context, it is easiest to understand and apply our results if the age classes are of some defined length, so we have added language to that effect (although the actual length is not important, so long as it is uniform).

*4. The reproductive schedule is one of the key aspects in the model. However, how different reproductive schedules are implemented in the computational model (assuming it’s in the Leslie matrix?) is not well explained, details of this in the Methods section will be helpful for readers to understand the modelling system.*

Thank you for this suggestion – we have revised the Methods under “Designating Reproductive and Epidemiological Variation” to be clearer about how the reproductive schedules provide fertility parameters for our models.

*5. Line 212 – “i_r declines drops” keep either “declines” or “drops”*

Thank you very much for catching this mistake; we have corrected it. *6. Figure 3A) Why the baseline value of i_r is different in the declining and rising cases? The message this figure conveys is the association between the change of infection risk and the optimal immune specificity under different reproductive schedules, it may be more convincing if these comparisons start with a same baseline value. The same concern applies to the results in Figure 4 as well.*

We understand the confusion and explored making a new version of the figures as suggested. However, ultimately we discovered that the proposed new version would confound two separate phenomena: 1) the change in infection risk with age (which we are especially interested in), and 2) the absolute level of risk experienced (which has been explored in prior publications and which we also feature in Fig. 3B). We became concerned that the proposed figure would make it difficult to disentangle the differing effects of these two and might lead to misattribution of the former effect to the latter. Please note that the particular point that we wish to highlight with Figures 3 and 4 is that, when the same infection risks are experienced but at different points in life, the relationship between reproductive schedule and optimal specificity is flipped. We have therefore kept the original figure, but we have altered the text in the results section to make this takeaway more clear.

*7. Line 230 – “The epidemiology is essential” This sentence is redundant – the point has been well made in the previous sentence.*

We have cut the sentence in question. *8. Results for Figure 4 are based on fixed parameter values of mu_d, mu_i, mu_id, pho, and gamma across 129 chordate species, which implicitly assumes that the immune responses for these species are the same. Is this assumption supported by any study? Because the optimal immune strategies could also be sensitive to these parameters. Why would you like to choose these particular values?*

This is an excellent point and an important place for us to offer some clarification in the text. We have now addressed this in the appropriate part of our Results section, as well as in the Methods, to make explicit that this is an assumption for simplicity’s sake, but that this not necessarily a likely case in nature. Furthermore, we did explore the impact of different values for *µ_i_*, *µ_d_*, etc., but found no qualitatively different conclusions from those generated by the values we used in the text. We now report that consistency in the Results section. Finally, because previous work by some of us has already described the influence of these parameter values on optimal sensitivity and specificity, we now refer to that previous work when justifying our parameter choices.

*9. Line 350 –“while when” – either will be fine*

We think it does not quite sound right to drop either one of these words without changing the preceding comma to a semicolon, so we have cut “while” and replaced the comma with a semicolon.

*10. Line 431 – “different their immune specificities” – “different immune specificities”*

Thank you very much for catching this error – we have corrected it.

*11. Overall the Discussion section is too long from my point of view. For example, paragraph discussing challenges and further benefits contain too many details. In particular, there are three “for example” in the paragraph discussing further benefits. These paragraphs can be shortened and combined.*	Trimmed as suggested. Please see further details of our response to this concern above.

*12. I find Figure S2 is a bit confusing. Are you trying to demonstrate the scaling of i_r, which is done by interpolating start and end points?*

We understand the confusion and have altered this figure to make the scaling process clearer. In particular, we have eliminated the first part of the figure, which demonstrated how not to scale *i_r_* for different lifespans; in retrospect, this seemed likely to add to confusion. We also have added a few lines of text to the figure legend to make the different steps more legible, while retaining a modified form of B) to illustrate the contrast between absolute rate of change (which does vary) and scaled rate of change (which we equalize through scaling).

*Reviewer #2: This study is a useful extension of prior models to investigate the evolution of immune specificity, assuming a tradeoff between sensitivity and specificity, i.e., pathology from infection or immunity. Several of the investigators had previously shown how age at reproduction could affect the optimal strategy. Here, the investigators examine how life-stage-varying infection risk interacts with life history to influence optimal specificity. Using a simple mathematical model, they show that changes in infection risk over a typical lifespan can completely flip the ranking of strategies (Fig 3). They then use empirical matrix population models from the COMADRE database to evaluate, given uncertainty in actual infection risk over time in different species, how the optimal specificity correlates with the mean reproductive rate and other demographic traits. They show again that life history alone is a poor predictor of specificity (Fig 4).*

*This is a careful analysis that should interest infectious disease, population, and evolutionary biologists and some evolutionarily minded immunologists. Host immune systems are under strong selective pressures, and yet there is little integrative theory that attempts to explain the diversity of strategies that are observed (or that probably would be observed, if we studied other species as well as we should). Although I like to think we will have a more nuanced understanding of actual immunological tradeoffs in a few decades, this is a logical start. The most important outcomes of this paper could be increased attention not only to potential tradeoffs, including immunopathology in other species, but also to the quantitative impact of pathogens on survival in different life stages.*

We thank the reviewer for their kind feedback on our manuscript.  *I have only minor suggestions for the paper, which presents a solid contribution in its current form. My primary suggestions are of the "but what about..." variety: What if pathogens' fitness impacts and/or immunopathology risk were mostly through fecundity rather than survival? How much could gamma and optimal specificity vary by life stage? Do infection risks with multigenerational or inconsistent periods (truly time-varying) change the optimal strategy? How does coevolution affect the optimal strategy? I am most curious about the first, although the Discussion is long enough, and life-stage-varying sensitivity is briefly mentioned.*

We agree that these are all very compelling points that merit addressing in our manuscript and thank the reviewer for raising them. Mindful of a suggestion from another reviewer that the Discussion is already too long, we did not explore them in great depth, but we have included at least a few sentences covering each, along with relevant citations. We address the first question in the portion of our discussion where we talk about elasticity of λ with respect to survival, by extending the analogy to take in elasticity of λ with respect to fertility. We respond to the questions about varying infection risk and coevolution together in a new paragraph devoted to considering the influence of parasite traits on our results. We also integrate a mention of Schnaack and Nourmohammad (2021) (as suggested by the Reviewer) in said paragraph, since we believe it is relevant to the question of co-evolution and the potential for there to be additional selective forces operating on specificity and sensitivity in the adaptive immune system, beyond those we explore with our model.

*My second general suggestion is that the authors consider providing more examples to support the existence of a tradeoff and potentially some background on population matrices. Few readers will be familiar with both areas.*

We appreciate the suggestions, which are helpful for identifying blindspots that we neglected. We have added material in the Introduction on both counts to better orient readers. There is a large body of literature detailing trade-offs between cross-reactivity and specificity in antibodies, though relatively little of it deals directly with immunopathology and whether or not an immune response is triggered, which is a part of our framework, so we have elected to not delve into it here. We have not made extensive additions to address this.

*My remaining suggestions involve possible clarifications and typos:

The authors may want to consider how Schnaack & Nourmohammad's results (eLife, 2021; https://elifesciences.org/articles/61346v1), which are effectively about sensitivity from immune memory, fit in with the conclusions.*

We appreciate the suggestion and agree that Schnaack and Nourmohammad’s results are very interesting and extremely relevant. As mentioned above, we have included a reference to their work in our Discussion section in the broader context of co-evolution of parasites. In their method, what they describe as “lifespan” is really a metric for the number of times an organism can expect to re-encounter a given parasite; this metric maps onto some combination of our demographic parameters and our infection risk, making the comparison with our concept of lifespan slightly tricky. For the sake of brevity we have elected not to explore this distinction in depth, although we think it is very interesting and offers some intriguing points for further consideration.

*ll. 385-399: R0 is often a better indicator than lambda of long-term competitive success, right? This paragraph suggests it is not important, but I find the passage confusing.*

For the sake of avoiding confusion, we have decided to delete the offending text from lines 395–399 that was the main issue. It is true to a certain extent that R_0_ can be a good indicator of long-term competitive success, and when density dependence affects o but Metcalf and Pavard (2008) suggest that R_0_ is best-suited to cases where generations are non-overlapping, whereas λ is also suitable for cases with overlapping generations because it is an increase per unit of time (in this case, per year, which nicely accords with the age class length we use in our COMADRE analysis).  *Figure 1: Panels B and C are reversed between the figure and caption.*

We very much appreciate this catch and have rectified our mistake.

*Figure 2: I found the horizontal and vertical lines in the right panel confusing. (I know a few other readers who felt the same way.) Why not just show points?*

We understand the concern, and we agree that showing points at the intersections on the trade-off curve helps significantly. We have decided to also retain the lines for two reasons. First, the figure looks very empty and unsatisfying without the lines, because there is a very large amount of white space between the trade-off curve with the points and the axes. Second, the lines make it easier visually to identify the relative differences in specificity and sensitivity between strategies by establishing position on the axes. Therefore we think that dropping the lines altogether would detract from the communicative capability of the figure. However, given the understandable confusion, we have altered the figure to A) include points on the trade-off curve as suggested and B) de-emphasize the lines somewhat so that they are clearly subordinate to the points. We have also edited the figure caption to clarify the roles of the points and lines.

*"Robusticity", not "robustness"?*

We have made the suggested change – it turns out that robusticity is a term generally restricted to anthropology, where one of us picked it up.

*Reviewer #3: This well written paper addresses theoretically the interesting question of what determines optimal immune investment and moreover why there is so much variation amongst hosts both within and between species. It makes an important contribution to this field examining in particular the relative investment in sensitivity and specificity of the immune system. It follows up on this framing of the problem by some of the previous authors in their Nature Comms paper and I think the characterization of sensitivity – how likely to fire the immune response – and specificity – how tightly aligned to the pathogen the response is – is very interesting to explore. Here they propose age structured models that include epidemiology – in the sense that they look at disease risk although they don’t have epidemiological feedbacks with a dynamical disease modeling approach – to examine how life-history and epi interact to determine variation in these traits. The use of the COMADRE demographic data set is very novel and important and they have a number of important results that help explain patterns in nature and will drive future research. The results in figure 3 are really interesting. This is exactly what you want theory like this to do. The usefulness of the approach is emphasized by the nice example they quote in the abstract of whether infection risk increases or decreases at maturity changes the sign of the selection on immune specificity. I think it is a good contribution and should be published.*
We thank the reviewer for their very kind and enthusiastic feedback.

*I have some detailed comments throughout that I outline in order below. Overall one criticism that I have is that it is not always clear that the paper is looking at a particular aspect of the immune response. One interesting about the paper is that it is looking at sensitivity and specificity, but at times immune response is used in very general terms. I think this can be a bit confusing for the reader as a lot of other work is focused on some sort of absolute amount of resistance or tolerance to infection. Also some readers may not equate immune response to sensitivity and specificity that you are addressing here. I understand why the authors want to be general, but I don’t think there is the need for such general language here. The sensitivity/specificity idea is central and very novel in itself and I think being clear about this would give the paper a bigger impact. It’s arguably more realistic and useful to be thinking about sensitivity/specificity rather than some absolute amount of immune response, so making this point clearly would be really useful I think.*

This is a very valuable suggestion; we appreciate that clarity on the focal aspects of immune response is very important and do want to avoid confusing our readers. We have gone through the text to change a number of our mentions of “immune strategy” and the like to “immune specificity and sensitivity,” so as to make clearer for the reader the specific nature of our inquiry. We have retained “immune strategy” for when we are speaking more generally (e.g. in the first few paragraphs of the introduction) and for a few instances just to remind readers that sensitivity and specificity are facets of immune strategy.

*My second overall comment is that it could be made clearer that epidemiology here is not looking at epidemiological feedbacks on evolution due to an underlying dynamical disease model. This is explicitly stated in the middle of the paper, but I think it would be useful to make this point earlier, particularly given the comparison to previous models – many of which have these feedbacks although some like Shudo et al and Hamilton et al don’t. I don’t see this as a problem, but I think it would make the comparison to previous work clearer.*

We agree that this is an important distinction to be made and one that was not adequately highlighted in our first submission. We have accordingly added text to raise this point A) early in the Introduction, when first covering epidemiology in immune strategy and B) when we describe the model framework late in the Introduction. We hope that this will more clearly orient the reader, particularly those familiar with the relevant literature, as suggested.

*Abstract

I didn’t find it that compelling that you could predict a priori zoonotic risky hosts or those at risk – you make some interesting arguments based on ideas of relative sensitivity and specificity of humans and zoonotic hosts, but I wouldn’t have these statements in the abstract. For me the paper makes a big contribution to our understanding of patterns seen in nature and the fact that you get these changes in the sign of selection is really important. I think your abstract should reflect those results.*

We appreciate the suggestion and have cut the text originally running from “species” in line 27 to the end of the abstract. We have added a little new text to emphasize the sign change, as recommended. This should keep the focus on the points the reviewer highlights as most important.

*Introduction

Line 48 – consider something different to “vast” share..*

We have substituted with the word “considerable” instead. *Line 55 – There is a lot of different terminology in the field and I was wondering why you used facultative rather than induced here. Are you trying to say different things or is it more being careful with language that is used in immunology in specific ways?*

We agree that the terminology can be confusing, and here we had no particular distinction between “facultative” and “inducible” in mind. Given that “inducible” is the dominant term used in theories of immune defense, and in particular is the term used in the two sources we cite for this sentence, we have substituted “inducible” here.

*Line 55 – it might be useful to start this sentence with “given immunopathology” or something as a reminder – some readers may not find this intuitive*

We appreciate the suggestion and agree that it improves clarity; we have made the change.

*Line 65 - this is a good description of previous work – but it may be good here to contrast explicitly eco(epi)- evo models with dynamical underlying disease models that examine ecological feedbacks and those that don’t – 9&10 don’t have the feedbacks – I think spending some more time clarifying that here would be useful to place this work in the field.*

We agree that it is good to highlight early that some models include ecological feedbacks with interesting outputs (as discussed above). We have therefore added a reference and highlighted that some, but not all, models take this approach. Given that references 9 & 10 address different features of immune strategy than 12 & 13, we thought it best not to delve into too much detail, but we have ensured that this distinction in models is addressed here.

*Line 95 – I think it is interesting to talk about herd immunity here, but more generalpoint is that prevalence is changed in these dynamical models even without acquired immunity*

We appreciate the point, which has also helped us better grasp the results of those papers. We have altered the text to capture the results and conclusions of these models more clearly and to establish their relevance to our work.

*Line 106 – the Cape Buffalo has specific data that make it stand out, but this general point of age related impacts could be more generally referenced*

It certainly is important to establish that this is a common phenomenon, not a limited one. References 38–40 do review, among other topics, a number of cases of age-based variation in burden and prevalence, so we altered our language to ensure that the general point was highlighted. And, while reviewing the literature around this point following the reviewer’s suggestion, we found another specific reference on Seychelles warblers documenting decreasing prevalence with age; given that it demonstrates a trend opposite that in the Cape buffalo, we included it as a useful contrast. *Line 131 – you use maximization of population growth rate as your fitness measure where as much o f the field is using ESS type resistance to invasion type analyses – I think it is important to explain this – you don’t have eco-evo feedbacks so it makes sense but it is worth explaining .*

We have inserted a few sentences highlighting this distinction and explaining why λ is a good fit for our particular framework and traits of interest.

*Line 136 – Background mortality can impact optimal immune defence even if risks don’t vary with age in other modeling frameworks – as you describe elsewhere – you should make it clear that this just applies to your approach.*

We appreciate the point and have included language that highlights that this result is specific to the aspect of immune strategy and the modeling framework that we are examining.

*Line 276 – I would consider not using the acronyms MRR, AFR and RLE – they are pretty informative spelled out and not used that much. Most readers are not going to know them.*

We have made the change; originally we used them for brevity and in our own notes, but, as pointed out, they don’t offer much value to readers. *Line 346 – using mutable may confuse some readers here*

We have substituted the word “changeable.” *Line 385. – Donnelly is looking at a different definition of resistance – I think it would be useful to discuss this.*

We agree that it’s important to make sure this distinction is understood. We have made a few changes to the text to ensure that our point is clearer. Our intention is just to draw an analogy (as noted in the text our framework is not tractable for the same type of analytical solution that Donnelly et al. (2015) were able to produce, so we cannot explicitly demonstrate that our results are of a piece). We have now explained the result from Donnelly et al. (2015) more clearly and highlighted the differences between our facet of immune strategy and theirs.  *Line 388 – Useful to explain ‘elasticity’ here.*

Thank you for noticing that we had not defined it clearly. Another reviewer wanted us to define it when it first appears in the Results section, so we have made that change, but we have also tidied some of the language in this section to remind readers about what elasticity is (including giving a formula) and why it is relevant to the analogy with Donnelly et al. (2015), as discussed above.
